# Prevalence, clinical features and prognosis of familial hypercholesterolemia in Chinese Han patients with acute coronary syndrome after a coronary event: a retrospective observational study

**DOI:** 10.1186/s12872-024-03803-4

**Published:** 2024-03-05

**Authors:** Huijuan Kou, Hongtao Wang, Peng Liu, Xin Wang, Wenjing Zhu, Wei Jiang, Xiaojun Hu, Jie Deng

**Affiliations:** 1 Department of Cardiology, The Second Affiliated Hospital, Xi’an Jiaotong University, Xi’an, Shaanxi P.R. China; 2 Department of Neurosurgery, Tangdu Hospital, Air Force Medical University, Xi’an, Shaanxi P.R. China

**Keywords:** Familial hypercholesterolemia, Cholesterol, Acute coronary syndrome, Coronary artery disease, Prognosis, MACCE

## Abstract

**Background:**

Familial hypercholesterolemia (FH) is an autosomal semi-dominant disease, characterized by markedly elevated levels of low-density lipoprotein cholesterol (LDL-c) from conception and accelerated atherosclerotic cardiovascular disease, often resulting in early death. The aim of this study was to evaluate the prevalence of clinically defined FH in Chinese Han patients with acute coronary syndrome (ACS) and compare the long-term prognosis of ACS patients with and without FH receiving lipid-lowering therapy containing statins after a coronary event.

**Methods:**

All ACS patients were screened at the Second Affiliated Hospital of Xi’an Jiaotong University between Jan 2019 and Sep 2020, and 531 participants were enrolled. All were examined for FH under the Dutch Lipid Clinical Network (DLCN) criteria, and those patients were divided into definite/probable FH, possible FH and unlikely FH. The severity of coronary artery disease was evaluated by the Gensini scoring system. Plasma levels of total cholesterol (TC), triacylglycerol (TG), HDL-cholesterol (HDL-c), LDL-cholesterol (LDL-c), very low-density lipoproteins-cholesterol (VLDL-c), apolipoprotein A1 (apoA1), apolipoprotein B (apoB) and lipoprotein (a) (Lp(a)) were determined centrally at baseline and the last follow-up visit in the fasting state. The non-high-density lipoprotein cholesterol (non-HDL-c) concentration, the TC/HDL-c and apoB/apoA1 ratios were calculated. After FH patients received lipid-lowering treatment containing statin, the target LDL-c levels recommended by the guidelines (LDL-c < 1.8 mmol/L or < 1.4 mmol/L and a reduction > 50% from baseline) were evaluated, and the occurrence of major adverse cardiovascular and cerebrovascular events (MACCE) during the 12-month follow-up was recorded.

**Results:**

The prevalence of clinically definite or probable FH was 4.3%, and the prevalence of possible FH was 10.6%. Compared with the unlikely FH patients with ACS, the FH patients had higher levels of TC, LDL-c, apoB, Lp(a), non-HDL-c, TC/HDL-c and apoB/apoA1 ratio, more severe coronary artery diseases and greater prevalence of left main and triple or multiple vessel lesions. After lipid-lowering therapy containing statins, a minority of FH patients reached the target LDL-c levels defined by the guidelines (χ^2^ = 33.527, *P* < 0.001). During the 12-month follow-up, a total of 72 patients experienced MACCE. The survival curve in patients in the FH group was significantly lower than that in the unlikely FH group (HR = 1.530, log-rank test: *P* < 0.05). Furthermore, the survival curve in patients with high LDL-c (≥ 1.8 mmol/L) was significantly lower than that in patients with low LDL-c (< 1.8 mmol/L) at the 12-month follow-up visit (HR = 1.394, log-rank test: *P* < 0.05). No significant difference was observed between patients with LDL-c levels ≥ 1.4 mmol/L and with < 1.4 mmol/L at the 12-month follow-up visit by using Kaplan–Meier survival analysis (HR = 1.282, log-rank test: *P* > 0.05).

**Conclusions:**

FH was an independent risk factor for MACCE in adult patients after a coronary event during long-term follow-up. However, there was inadequate high-intensity statins prescriptions for high-risk patients in this current study. It is important for FH patients to optimize lipid-lowering treatment strategies to reach the target LDL-c level to improve the long-term prognosis of clinical outcomes.

**Supplementary Information:**

The online version contains supplementary material available at 10.1186/s12872-024-03803-4.

## Introduction

Familial hypercholesterolemia (FH) is an autosomal semi-dominant disease, characterized by markedly elevated levels of low-density lipoprotein cholesterol (LDL-c) from conception and accelerated atherosclerotic cardiovascular disease, often resulting in early death if undiagnosed and untreated promptly [1–3]. FH may affect up to 35 million people worldwide, but only 10% are currently diagnosed, and > 80% of those treated do not achieve recommended LDL-c goals [2, 4].

Although FH is known to result from deleterious mutations in genes correlated with the LDL receptor pathway, mainly LDL receptor (LDLR), apolipoprotein B (APOB), proprotein convertase subtilisin/kexin type 9 (PCSK9) and low-density lipoprotein receptor adaptor protein 1 (LDLRAP1) [5, 6], genetic testing has seldom been utilized in clinical settings. The Dutch Lipid Clinical Network (DLCN) scoring system, as the internationally recognized diagnostic criteria for FH, is one of the most valuable evaluation methods to assess the phenotype of FH [1, 2, 6].

Dyslipidemia in FH, particularly elevated LDL-c concentrations, can promote the progression of atherosclerotic diseases and increase the risk of premature coronary artery disease morbidity and mortality. Therefore, it is important to accurately diagnose and screen FH in patients with ACS. Long-term lipid-lowering therapy can reduce the burden of atherosclerotic cardiovascular disease in high-risk FH patients with ACS [7–10], but few FH patients can achieve the target LDL-c level of < 1.8 mmol/L and a reduction > 50% from baseline. In addition, FH is an independent risk factor for major adverse cardiovascular and cerebrovascular events (MACCE) in patients after a coronary event during long-term follow-up [8, 11–13]. Thus, it is necessary to optimize the lipid-targeting treatment of patients with FH after a coronary event [2, 7–9, 11].

In this retrospective observational study, we evaluated the prevalence of clinically defined FH in patients with ACS and compare the clinical features and long-term prognosis of those with and without FH who were on lipid-lowering therapy containing statin after a coronary event.

## Methods

### Subjects

All subjects were consecutively recruited at the Second Affiliated Hospital of Xi’an Jiaotong University between Jan 2019 and Sep 2020. All patients presenting with acute coronary syndrome (ACS) and receiving the invasive angiography for coronary revascularization were eligible. The ACS definition was based on the 2023 ESC Guidelines for the Management of ACS [14]. All enrolled patients were older than 18 and took no lipid-lowering treatment within 3 months before admission. Baseline demographics, cardiovascular risk factors, and family history were collected by a trained doctor from the medical records. Plasma cholesterol levels were measured within 24 h of admission to the hospital. Patients were excluded if blood lipid data were missing or in the case of pregnancy, infectious or systematic inflammatory disease, significant hematologic disorders, thyroid dysfunction, severe liver or renal dysfunction, or malignant tumors.

### Diagnostic criteria for FH

All patients were evaluated for familial hypercholesterolaemia (FH) based on the Dutch Lipid Clinical Network (DLCN) criteria [1, 15, 16], including family history, clinical history, physical examination, LDL-c levels and DNA analysis if available. Patients were categorized as definite FH (DLCN score > 8), probable FH (DLCN score 6–8), possible FH (DLCN score 3–5) and unlikely FH (DLCN score < 3) [1, 15, 16]. Notably, family history of elevated LDL-c was not available for our study sample, so we scored this item as ‘0’ in the DLCN algorithm.

### Assessment of the severity of coronary lesions

We evaluated the coronary severity of each patient by the Gensini scoring system. The Gensini score (GS) was computed by assigning a severity score to each coronary stenosis according to the degree of luminal narrowing and its geographic importance. First, reductions in the lumen diameter or roentgenographic appearances of the coronary lesion were evaluated as 1 for 1–25% stenosis, 2 for 26–50% stenosis, 4 for 51–75% stenosis, 8 for 76–90% stenosis, 16 for 91–99% stenosis and 32 for total occlusion. These scores were multiplied by the weight coefficient that represented the importance of the lesion’s position: 5 for the left main coronary artery, 2.5 for the proximal left anterior descending or proximal left circumflex artery, 1.5 for the mid-region, 1 for the distal left anterior descending or mid-distal region of the left circumflex artery or proximal‒distal right coronary artery, and 0.5 for small vascular branches.

### Study design

This retrospective observational study adhered to the principles of the Declaration of Helsinki, and the investigational protocol was approved by the Ethics Committee for Human Studies at the Second Affiliated Hospital of Xi’an Jiaotong University. Written informed consent was obtained from all patients who enrolled into the study.

**Fig. 1:**
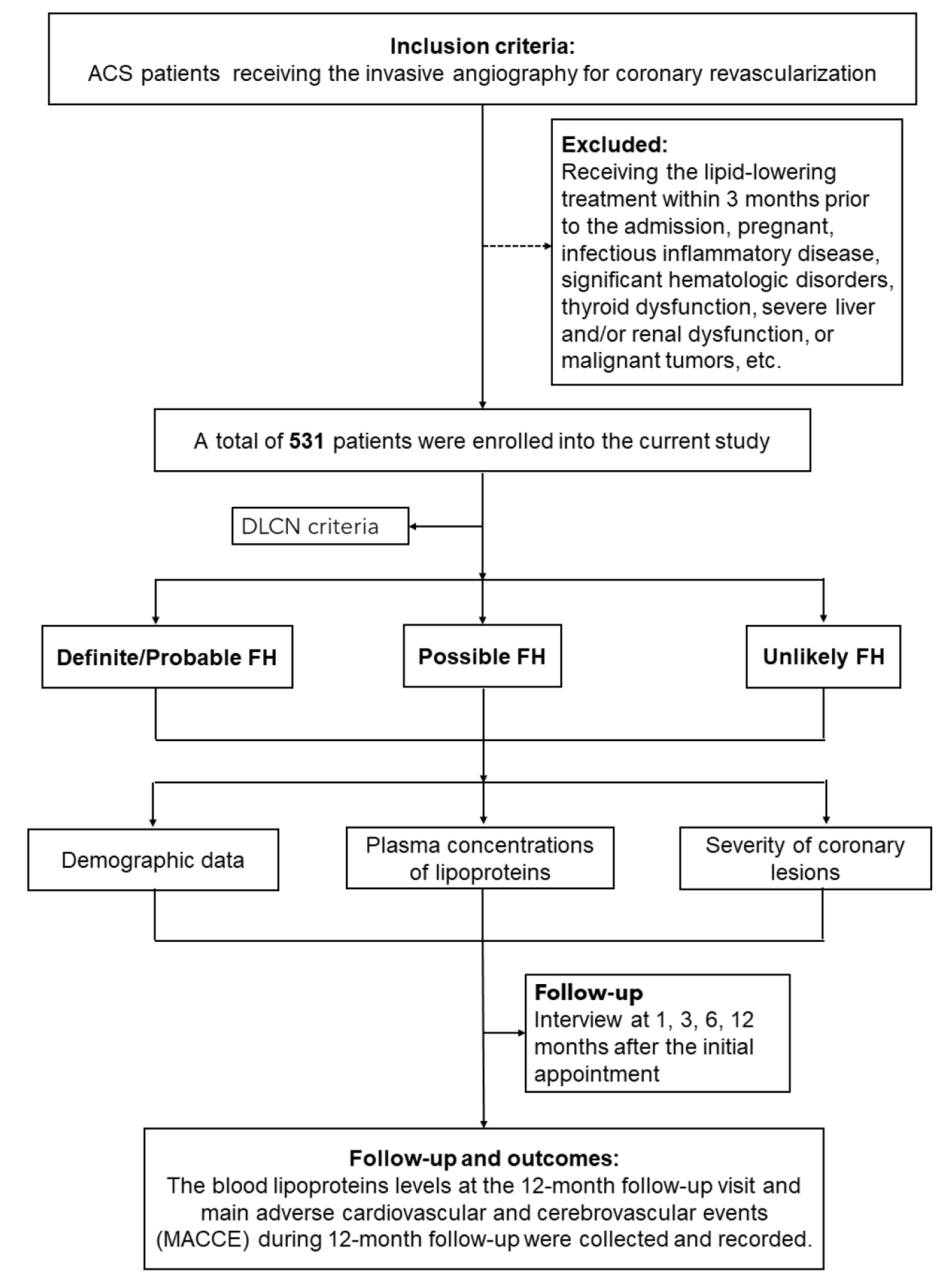
Diagram of enrolled participants and follow-up outcomes

A total of 1083 subjects receiving invasive angiography were screened, and 531 patients were ultimately enrolled. According to the DLCN criteria [1, 15, 16], the FH patients were divided into 3 groups: definite/probable FH, possible FH and unlikely FH. Demographic data, such as age, sex, alcohol intake, cigarette smoking, and family histories of hypertension, diabetes mellitus and coronary artery diseases, were obtained from all participants. Body mass index (BMI) was calculated as weight (kg)/height^2^ (m^2^). Blood pressure measurements, including systolic (SBP) and diastolic blood pressure (DBP) measurements, were taken from medical records (Fig. 1).

### Assessments of plasma concentrations of lipoproteins

Plasma concentrations of total cholesterol (TC), triacylglycerol (TG), high-density lipoprotein cholesterol (HDL-c), LDL-cholesterol (LDL-c), very low-density lipoproteins-cholesterol (VLDL-c), apolipoprotein A1 (apoA1), apolipoprotein B (apoB) and lipoprotein (a) (Lp(a)) were determined centrally at baseline and the last follow-up visit in the fasting state using an enzymatic colorimetric method and running on an automated analyzer (Cobas 8000 c 701, Roche, Germany). The non-high-density lipoprotein cholesterol (non-HDL-c) concentration was calculated as the TC value minus HDL-c [17]. The TC/HDL-c and apoB/apoA1 ratios were calculated [18–20].

### Follow-up and outcomes

After an ACS event, lipid-lowering treatment should be initiated with a high-intensity statins (e.g. atorvastatin or rosuvastatin) as early as possible. In clinical practice, load dose statins (atorvastatin 40-80 mg or rosuvastatin 20 mg) were prescribed to acute myocardial infarction patients during the perioperative period to further reduce major cardiovascular events. According to the risk stratification, some high-risk patients received high dose high-intensity statins (atorvastatin 40-80 mg/d or rosuvastatin 20 mg/d) and/or a combined medication strategy (e.g. high-intensity statins plus ezetimibe or fenofibrate or others). It is recommended that the target LDL-c level was < 1.8 mmol/L and > 50% reduction from baseline in adult patients with FH. The current treatment goal for secondary prevention is to lower LDL-C to < 1.4 mmol/L and to achieve a ≥ 50% LDL-C reduction from baseline for high-risk FH patients [14–16]. All the patients were followed up at 1, 3, 6 and 12 months by telephone and/or in person after hospital admission. The primary outcome was major adverse cardiovascular and cerebrovascular events (MACCE), which were defined as cardiac death, acute myocardial infarction (AMI), acute decompensated heart failure requiring hospitalization, cerebrovascular events or ischemia-driven revascularization. Cardiac death was primarily confirmed by death from cardiac causes, including sudden cardiac death, congestive heart failure, AMI, severe arrhythmia, stroke, or other structural/functional cardiac diseases. AMI was diagnosed by a comprehensive evaluation combining chest pain or equivalent symptom complex, diagnostic changes in cardiac enzyme levels, and electrocardiogram. The definition of stroke was acute cerebral infarction on the basis of imaging or typical symptoms. Ischemia-driven revascularization was defined as repeated percutaneous coronary intervention or coronary artery bypass grafting of lesions in the presence of AMI, unstable or stable angina, or documented silent ischemia.

### Statistical analysis

Statistical analyses were performed with Statistical Product and Service Solutions for Windows (SPSS, version 19.0). Variables were given as mean (standard deviation), median (interquartile range) or n (%), as appropriate. Normally distributed values were analyzed using the Kolmogorov‒Smirnov test. Categorical variables were analyzed with chi-squared test or Fisher’s exact test, and continuous variables were analyzed with Mann‒Whitney U tests, t tests, the Kruskal‒Wallis H test or one-way ANOVA. Differences in baseline characteristics among the 3 groups were analyzed with chi-squared test or the Kruskal‒Wallis H test (for continuous variables that were not normally distributed). After adjusting for traditional covariates, hazard ratios (HRs) and their 95% confidence intervals (CIs) were calculated using a logistic multivariable model. Event-free survival was analyzed using the Kaplan‒Meier method, and intergroup differences in survival were assessed for significance using the log-rank test. For all tests, *P* < 0.05 was considered statistically significant.

## Results

### Baseline demographic characteristics and blood lipid profiles of all enrolled patients

A total of 531 patients receiving invasive angiography for coronary revascularization were enrolled and were divided into 3 groups based on the DLCN criteria. Among all the participants, 15 (4.3%) had a definite/probable FH phenotype, and 56 (10.6%) had a possible FH phenotype. The other 460 (86.6%) had DLCN scores of “unlikely FH phenotype” and so were classified as not having FH. No differences in sex distribution, smoking status, drinking status, history of hypertension, diabetes mellitus or coronary artery diseases were observed among the 3 groups. Compared with patients without FH, those with definite/probable and possible FH were younger but showed higher TC (8.29, 5.54 vs. 3.76 mmol/L, *P* < 0.001), LDL-c (5.62, 4.07 vs. 2.29 mmol/L, *P* = 0.001), apoB (0.95, 0.95 vs. 0.84 g/L, *P* = 0.004), Lp(a) (29.0, 19.8 vs. 11.3 mg/dL, *P* = 0.007), non-HDL-c (7.09, 4.47 vs. 2.70 mmol/L, *P* < 0.001), TC/HDL-c ratio (7.67, 5.37 vs. 3.63, *P* < 0.001) and apoB/apoA1 ratio (0.86, 0.73 vs. 0.65, *P* < 0.001) (Table 1).

**Table 1:** Baseline demographic characteristics and blood lipid profiles

		The diagnostic probability of FH phenotype				
Variable	All	Definite/Probable	Possible	Unlikely	χ2/U	*P* value
**Number, n(%)**	531(100%)	15(2.82%)	56(10.55%)	460(86.63%)		
**Demographics**						
Age, yrs	61(54, 67)	48(41, 56)	53(47, 55)	62(56, 67)	64.660	**< 0.001**
Female, n(%)	115(21.7)	5(33.3)	15(26.8)	95(20.7)	2.347	0.309
BMI, Kg/m^2^	24.62(23.18, 26.67)	24.19(22.74, 26.18)	25.95(24.22, 27.85)	24.49(22.92, 26.56)	10.240	**0.006**
Smoking, n(%)	244(46.0)	5(33.3)	28(50.0)	211(45.9)	1.332	0.521
Alcohol, n(%)	137(25.8)	4(26.7)	13(23.2)	120(26.1)	0.242	0.886
Hypertension, n(%)	284(53.5)	8(53.3)	33(58.9)	243(52.8)	0.747	0.688
Diabetes mellitus, n(%)	124(23.5)	2(13.3)	12(21.4)	111(24.1)	0.821	0.679
Pre-existing CAD, n(%)	47(9.0)	2(13.3)	6(10.7)	40(8.7)	1.115	0.530
**Lipid profiles**						
TC, mmol/L	3.86(3.22, 4.67)	8.29(7.29, 8.98)	5.54(3.84, 6.43)	3.76(3.18, 4.41)	81.928	**< 0.001**
TG, mmol/L	1.43(1.07, 1.88)	1.35(0.89, 1.58)	1.79(1.31, 2.20)	1.40(1.06, 1.82)	13.020	**0.001**
HDL-c, mmol/L	1.01(0.88, 1.19)	0.99(0.95, 1.04)	1.01(0.87, 1.16)	1.01(0.88, 1.20)	0.335	0.846
LDL-c, mmol/L	2.40(1.85, 3.05)	5.62(5.12, 5.99)	4.07(2.23, 4.45)	2.29(1.78, 2.82)	78.454	**< 0.001**
VLDL, mmol/L	0.35(0.19, 0.58)	0.33(0.10, 0.53)	0.52(0.37, 0.73)	0.33(0.19, 0.55)	14.282	**0.001**
ApoA1, g/L	1.26(1.10, 1.46)	1.14(0.88, 1.32)	1.25(1.07, 1.50)	1.27(1.11, 1.46)	4.541	0.103
ApoB, g/L	0.85(0.69, 1.01)	0.95(0.86, 1.02)	0.95(0.75, 1.23)	0.84(0.69, 0.98)	11.099	**0.004**
Lp(a), mg/dL	12.0(4.9, 29.6)	29.0(17.7, 35.1)	19.8(5.1, 38.5)	11.3(4.8, 26.8)	9.810	**0.007**
Non-HDL-c, mmol/L	2.78(2.19, 3.55)	7.09(6.14, 7.94)	4.47(2.82, 5.40)	2.70(2.16, 3.32)	83.053	**< 0.001**
TC/HDL-c	3.75(3.06, 4.78)	7.67(6.17, 9.13)	5.37(3.67, 6.10)	3.63(2.98, 4.38)	73.943	**< 0.001**
ApoB/ApoA1	0.67(0.54, 0.82)	0.86(0.66, 1.07)	0.73(0.58, 0.99)	0.65(0.52, 0.80)	16.384	**< 0.001**

FH: Family hypercholesterolaemia; BMI: Body mass index; CAD: Coronary artery disease; TC: Total cholesterol; TG: Triglyceride; HDL-c: High-density lipoprotein cholesterol; LDL-c: Low-density lipoprotein cholesterol; VLDL: Very low-density lipoprotein

### Clinical features, angiographic characteristics, and medical treatment therapies in the different groups

As shown in Table 2, non-ST-segment elevation myocardial infarction (NSTEMI) was a common cause of hospitalization in possible FH patients (23.2%), and the difference in this rate among the 3 groups was statistically significant (*P* = 0.022). Based on the results of coronary angiography, single-vessel lesions were common in patients without FH (*P* = 0.001), while triple- or multiple-vessel lesions (*P* < 0.001) and left main diseases (*P* = 0.010) were common in patients with FH. Patients with the FH phenotype had a higher GS than those without FH (42, 54 vs. 36, *P* < 0.001). In the study, we found that impossible and possible FH patients were prescribed to the statin monotherapy predominantly (94.1% and 89.3% vs. 46.7%, *P* < 0.001). However, patients with definite or probable FH were prescribed to large dose high-intensity statins (53.3%) or a combination of high-intensity statins and ezetimibe (26.7%) (*P* < 0.001). Notably, 2 patients with a definite FH phenotype were diagnosed with heterozygous FH after completing the genetic test and received the targeted PCSK9 inhibitor together with statin therapy.

**Table 2:** Clinical features, angiographic characteristics, and medical treatment therapy at the time of discharge for the first hospitalization

		The diagnostic probability of FH phenotype				
Variable	All	Definite/Probable	Possible	Unlikely	χ2/U	*P* value
**Number, n(%)**	531(100%)	15(2.82%)	56(10.55%)	460(86.63%)		
**Diagnosis**						
UAP, n(%)	277(52.2)	6(40.0)	24(42.9)	247(53.7)	3.266	0.198
STEMI, n(%)	191(36.0)	7(46.7)	19(33.9)	165(35.9)	0.849	0.669
NSTEMI, n(%)	63(11.9)	2(13.3)	13(23.2)	48(10.4)	7.178	**0.022**
**Coronary angiography**						
Single vessel, n(%)	207(39.0)	3(20.0)	10(17.9)	194(42.2)	14.749	**0.001**
Double vessel, n(%)	176(33.1)	4(26.7)	20(35.7)	152(33.0)	0.427	0.855
Triple or multiple vessel lesions, n(%)	148(27.9)	8(53.3)	26(46.4)	114(24.8)	15.608	**< 0.001**
Left main, n(%)	34(6.4)	3(20.0)	7(12.5)	24(5.2)	8.454	**0.010**
Gensini score	38(24, 61)	42(28, 96)	54(32, 98)	36(20,56)	25.024	**< 0.001**
**Medical treatment**						
DAPT, n(%)	531(100.0)	15(100.0)	56(100.0)	460 (100.0)		
β- blocker, n(%)	197(37.1)	8(53.3)	20(35.7)	169(36.7)	1.766	0.430
ARNI/ACEI/ARB, n(%)	74(13.9)	4(26.7)	10(17.9)	60(13.0)	3.347	0.162
CCB, n(%)	116(21.8)	5(33.3)	15(26.8)	96(20.9)	2.487	0.279
High-intensity statin monotherapy, n(%)	490(92.3)	7(46.7)	50(89.3)	433(94.1)	25.764	**< 0.001**
High dose high-intensity statin therapy, n(%)	41(7.7)	8(53.3)	6(10.7)	27(5.9)	25.764	**< 0.001**
Combination with ezetimibe, n(%)	17(3.2)	4(26.7)	3(5.4)	10(2.2)	15.380	**< 0.001**
Combination with fenofibrate, n(%)	16(3.0)	1(6.7)	2(3.6)	13(2.8)	0.800	0.670
Antidiabetic drugs, n(%)	80(15.1)	4(26.7)	9(16.1)	67(14.6)	1.985	0.379

FH: Family hypercholesterolaemia; UAP: Unstable angina pectoris; STEMI: ST-elevated myocardial infarction; NSTEMI: Non-ST-elevated myocardial infarction; DAPT: Double antiplatelet therapy; ARNI: Angiotensin receptor-neprilysin Inhibitor; ACEI: Angiotensin-converting enzyme inhibitor; ARB: Angiotensin receptor blocker; CCB: calcium channel blocker

High-intensity statin monotherapy: atorvastatin 20 mg/d or rosuvastatin 10 mg/d

High dose high-intensity statin therapy: atorvastatin 40-80 mg/d or rosuvastatin 20 mg/d

### Outcomes of enrolled patients with ACS based on the FH phenotype

During the 12-month follow-up, 72 patients (13.5% of all patients followed up) experienced MACCE, including ischemia-driven revascularization in 50 cases, cerebrovascular events in 12 cases, acute myocardial infarction in 3 cases, acute decompensated heart failure requiring hospitalization in 5 cases, and cardiac death in 2 cases. Univariate analysis showed that patients with a definite/probable or possible FH phenotype had a 7.282-fold higher risk of MACCE than those without FH (HR = 7.282, 95% CI = 4.127–12.849, χ^2^ = 57.574, *P* < 0.001). In a logistic multivariable model adjusted for some traditional covariates, such as age, sex, BMI, FH phenotype, smoking and alcohol status, history of hypertension and diabetes mellitus, severity of coronary artery diseases and blood lipoproteins levels, FH patients had 9.174-fold the risk of MACCE of patients without FH phenotype (HR = 9.174, 95% CI = 3.436–24.390, *P* < 0.001), male patients had 3.249-fold the risk of MACCE of female patients (HR = 3.249, 95% CI = 1.307–8.078, *P* = 0.011), smoking patients had 2.192-fold the risk of MACCE of non-smoking patients (HR = 2.192, 95% CI = 1.109–4.335, *P* = 0.024), patients with elevated TC concentrations were at 12.823-fold the risk of MACCE of the control group (HR = 12.823, 95% CI = 1.479–111.171, *P* = 0.021), patients with higher non-HDL-c were at 7.299-fold the risk of MACCE of the control group (HR = 7.299, 95% CI = 1.171–45.454, *P* = 0.033), and patients with elevated Lp(a) were at 1.012-fold the risk of MACCE of the control group (HR = 1.012, 95% CI = 1.001–1.022, *P* = 0.027).

### Kaplan–Meier curve for patients with or without FH

**Fig. 2:**
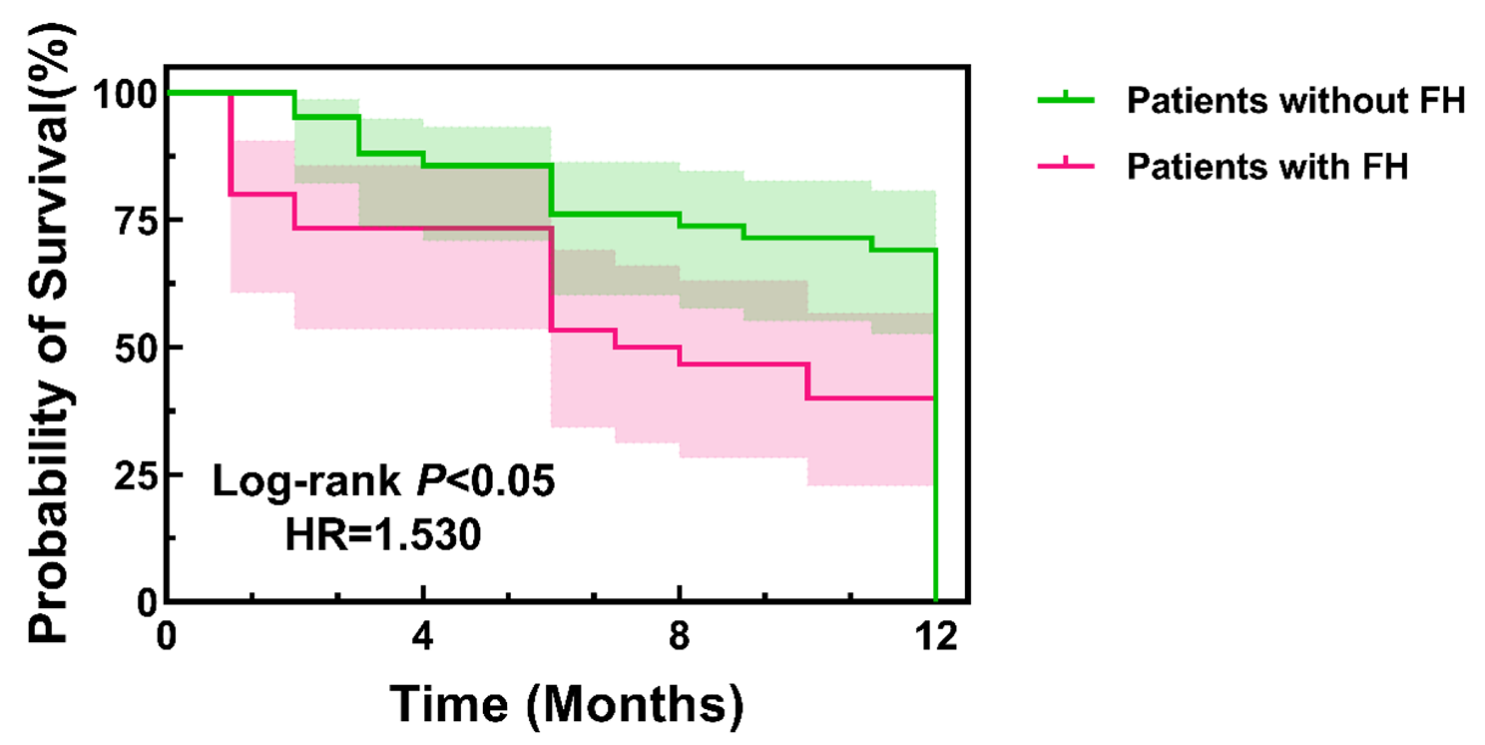
Kaplan–Meier survival curve for patients with or without the FH phenotype

A total of 72 patients had MACCE during the 12-month follow-up. Thirty patients with the FH phenotype, including definite/probable and possible FH, and 42 patients without FH experienced MACCE. The Kaplan–Meier survival curve in patients in the FH group was significantly lower than that in the unlikely FH group (HR = 1.530, log-rank test: *P* < 0.05) (Fig. 2).

The survival probability of patients with the FH phenotype is indicated by the solid red line, and that of patients in the unlikely FH group is indicated by the solid green line. The survival curve in patients in the FH group was significantly lower than that in the unlikely FH group (HR = 1.530, log-rank test: *P* < 0.05). FH: familial hypercholesterolaemia; HR, hazard ratio.

### Kaplan–Meier curve for patients with different LDL-c levels at the 12-month follow-up visit

**Fig. 3:**
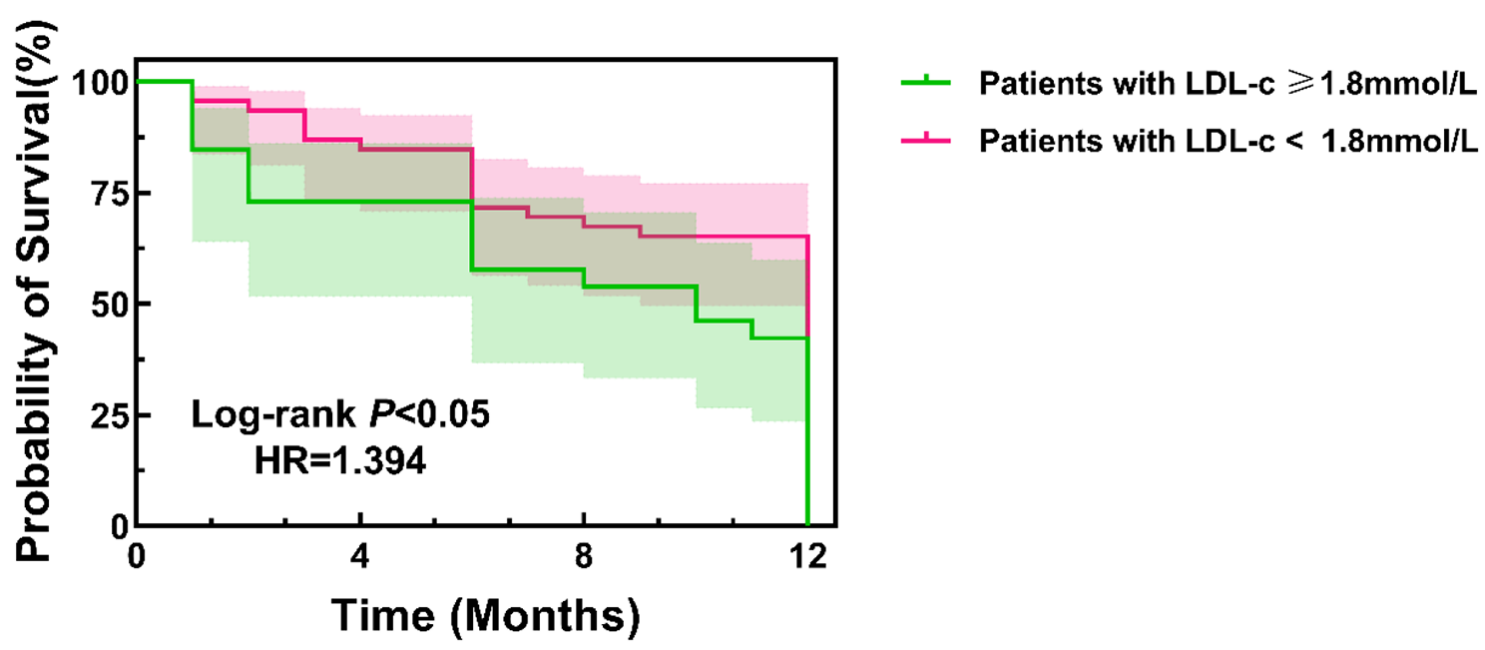
Kaplan–Meier survival curve for patients with different LDL-c levels (LDL-c ≥ 1.8 or < 1.8 mmol/L) at the 12-month follow-up visit

The blood lipoprotein levels of all patients enrolled in the study were collected at the 12-month follow-up visit. A total of 350 patients with LDL-c < 1.8 mmol/L were observed, 2 of whom were in the definite/probable FH group, 25 in the possible FH group and 323 in the unlikely FH group. The Kaplan–Meier survival curve of patients with high LDL-c was significantly below than that of patients with low LDL-c at the 12-month follow-up visit (HR = 1.394, log-rank test: *P* < 0.05) (Fig. 3).

The survival probability of patients with low LDL-c at the 12-month follow-up visit (LDL-c < 1.8 mmol/L) is indicated by the solid red line, and that of patients with high LDL-c at the 12-month follow-up visit (LDL-c ≥ 1.8 mmol/L) is indicated by the solid green line. The survival curve in patients with high LDL-c levels was significantly lower than that in patients with low LDL-c at the 12-month follow-up visit (HR = 1.394, log-rank test: *P* < 0.05). LDL-c: LDL cholesterol; HR, hazard ratio.

**Fig. 4:**
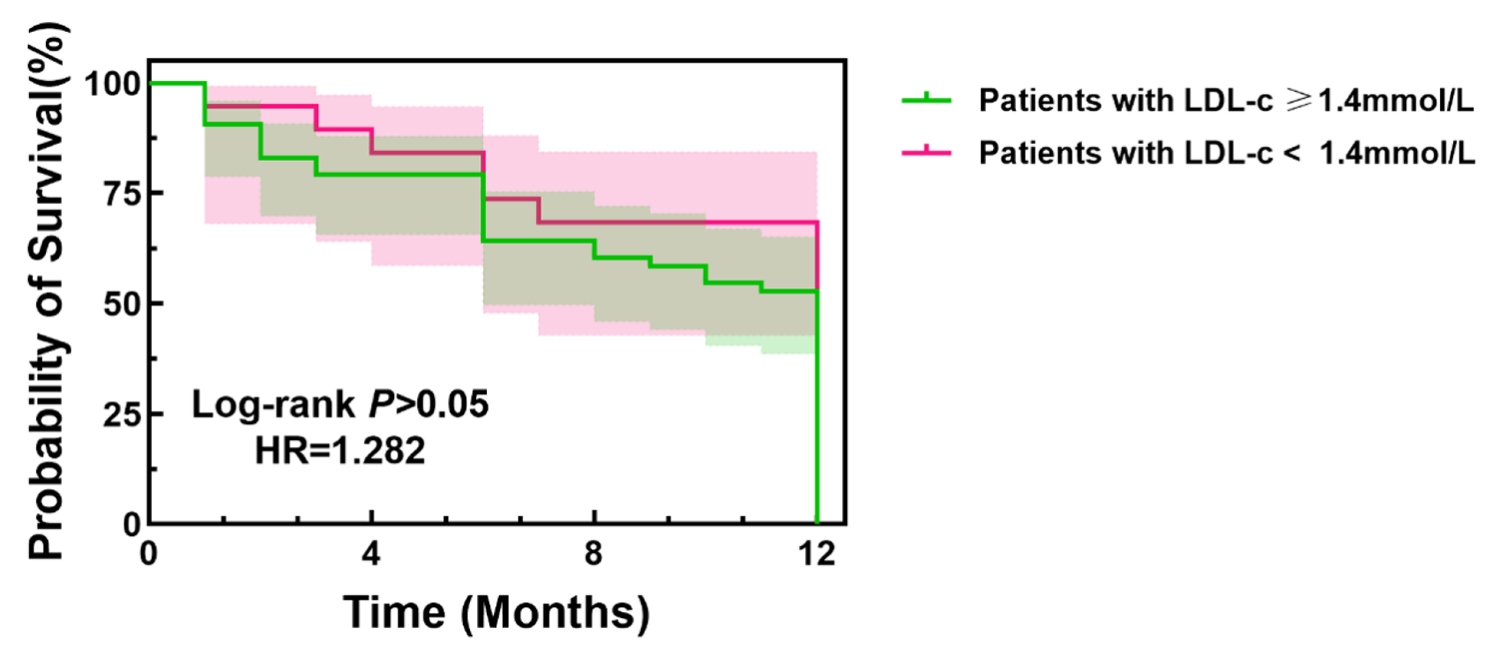
Kaplan–Meier survival curve for patients with different LDL-c levels (LDL-c ≥ 1.4 or < 1.4 mmol/L) at the 12-month follow-up visit

The blood lipoprotein levels of all patients enrolled in the study were collected at the 12-month follow-up visit. A total of 167 patients with LDL-c < 1.4 mmol/L were observed, 1 of whom was in the definite/probable FH group, 7 in the possible FH group and 159 in the unlikely FH group. No significant difference was observed between patients with different LDL-c levels (LDL-c ≥ 1.4 or < 1.4 mmol/L) at the 12-month follow-up visit by using Kaplan–Meier survival analysis (HR = 1.282, log-rank test: *P* > 0.05) (Fig. 4).

The survival probability of patients with low LDL-c at the 12-month follow-up visit (LDL-c < 1.4 mmol/L) is indicated by the solid red line, and that of patients with high LDL-c at the 12-month follow-up visit (LDL-c ≥ 1.4 mmol/L) is indicated by the solid green line. LDL-c: LDL cholesterol; HR, hazard ratio.

## Discussion

The present study demonstrated that the prevalence of clinically definite or probable FH was 4.3% and that the prevalence of possible FH was 10.6% in all the participants, based on the DLCN algorithm. Compared with the unlikely FH patients, FH patients with ACS had higher levels of TC, LDL-c, apoB, Lp(a), non-HDL-c, TC/HDL-c and apoB/apoA1 ratio and had more serious coronary diseases and greater prevalence of left main, triple-, or multiple-vessel lesions at the time of discharge. After an ACS event, lipid-lowering treatment should be initiated as early as possible, both for prognostic benefit and to increase patient adherence after discharge. Despite being prescribed high-intensity statins and/or a combined lipid-lowering therapy containing statins, a minority of FH patients reached the target LDL-c levels defined by the guidelines. After the Kaplan–Meier survival analysis, FH was an independent risk factor for MACCE in adult patients after a coronary event during long-term follow-up. Moreover, patients with high LDL-c (≥ 1.8 mmol/L) had significantly lower probability of survival than those with low LDL-c (< 1.8 mmol/L) at the 12-month follow-up visit. Therefore, it is very important for FH patients to optimize lipid-lowering treatment strategies to reach the target LDL-c level to improve the long-term prognosis of clinical outcomes.

As a hereditary disease, FH is an important cause of premature coronary artery disease. According to the DLCN FH criteria, we can identify the FH phenotype rapidly but without high cost. Haskiah et al [8] first highlighted the issue of FH among young Israeli adults who experienced first-time ACS and found that the prevalence of clinically defined FH in this population was 8.9%. A single-center study in Japan reported 100 (14.7%), 57 (8.4%), 156 (22.9%), and 367 (54.0%) subjects out of a total of 680 dyslipidemic participants were diagnosed as having definite, probable, possible, and unlikely FH by the DLCN FH criteria, respectively [13]. A European multi-country study involving 1451 patients with premature ACS and younger than 55 years (men) or 60 years (women) reported a prevalence of 4.8% for definite/probable FH and 47.1% for possible FH [21]. Our findings indicated that the prevalence of clinically definite or probable FH was 4.3% and possible FH was 10.6% in all the participants based on the DLCN algorithm, which is partially consistent with these recent studies. Since lipid levels are known to decrease during acute events, the consequences bias of the LDL-c levels at baseline should be concerned closely in the following clinical study. It should be noted that family history of elevated LDL-c was not available for all participants, so this item was entered as ‘0’ in the DLCN algorithm. With 2 exceptions, the other FH patients did not undergo genetic testing. Thus, the scores of DNA analysis were most often scored ‘0’.

Dyslipidemia was clearly observed in the FH patients. Several new markers have been introduced as alternative means to refine risk estimation beyond LDL-c in the presence of cardiovascular disease, such as non-HDL-c, TC/HDL-c ratio, and the apoB/apoA1 ratio [18, 22]. Unlike LDL-c, non-HDL-C refers to the cholesterol content found in all lipoproteins that contribute to atherosclerosis. Therefore, subtracting HDL-c from TC yields the non-HDL-C value, which represents the cholesterol carried by all lipoproteins except HDL-c [17]. The plasma apoB level is approximately equal to the sum of triglyceride-rich very-low-density lipoprotein, cholesterol-rich LDL, and Lp(a), representing the number of circulating atherogenic particles [23]. Several epidemiological studies and clinical trials have suggested that high apoB concentration, low apoA1 concentration and the apoB/apoA1 ratio may be better markers for the risk of coronary vascular disease than LDL-c and the TC/HDL-c ratio. The apoB/apoA1 ratio partially reflects the cholesterol balance between potentially atherogenic and anti-atherogenic lipoprotein particles and has been a useful predictor of cardiovascular events [24–26]. Lp(a) is a LDL-like particle composed of apolipoprotein B100 but with distinctive physiological effects. A significantly elevated level of Lp(a) is an important predictive variable for CHD risk in patients with FH [27–31]. In the present study, along with elevated LDL-cholesterol, several blood lipid profiles, including non-HDL-c, apoB, Lp(a), TC/HDL-c and apoB/apoA1 ratio, were significantly increased and were associated with the severity of coronary artery disease in FH patients.

FH not only can accelerate the occurrence of dyslipidemia but also can promote the progression of atherosclerotic diseases in ACS patients. Wang et al [32] demonstrated that the prevalence of molecularly defined FH in their enrolled patients was 26.9%, and coronary artery lesions were more severe in patients with FH than in those without. After lipid-lowering therapy, patients with FH still had significantly higher LDL-c at their last visit than those without. FH is associated with an increased risk of cardiovascular events in ACS and is an independent risk factor for ACS. In the present study, we also found that the enrolled ACS patients with definite/probable or possible FH exhibited more severe coronary atherosclerosis (*P* = 0.022), higher GS (*P* < 0.001) and a higher prevalence of left main (*P* = 0.010) and ≥ 3-vessel lesions (*P* < 0.001) than those without FH. Furthermore, FH patients received powerful lipid-lowering treatments, including large dose high-intensity statins and/or combination treatment with statins plus ezetimibe, compared to those without FH. However, few FH patients achieved optimal LDL-c levels at the 12-month follow-up visit.

As reported by Haskiah et al., approximate 18.0% and 11.5% of patients with FH attained their target LDL-c levels of < 70 and < 55 mg/100 mL at 1 year, respectively, despite impressive reductions in median absolute and relative levels and the fact that 85% of these patients were prescribed high-intensity statins at the time of discharge [8]. In this study, it is demonstrated that only 13.33% of the patients with definite/probable FH and 44.64% of these patients with possible FH achieved optimal LDL-c of < 1.8 mmol/L and > 50% reduction from baseline after receiving lipid-lowering treatment during the 12-month follow-up after a coronary event. Furthermore, merely 6.67% of the patients with definite/probable FH and 12.5% of these patients with possible FH achieved optimal LDL-c of < 1.4 mmol/L and > 50% reduction from baseline after receiving lipid-lowering treatment during the 12-month follow-up after a coronary event. However, there was inadequate high-intensity statins prescriptions for high-risk patients in this current study. We found that only 7.7% of patients were receiving the high dose high-intensity statins (atorvastatin 40-80 mg/d or rosuvastatin 20 mg/d) and 3.2% of patients were prescribed to a combined lipid-lowering therapy containing statins plus ezetimibe. The reasons need to be considered: some patients failed to adhere to long-term statin monotherapy or high dose high-intensity lipid-lowering therapy or a combined medication strategy due to the statin-associated risk of elevated liver enzymes and creatine kinase.

It was reported that long-term persistent lipid-lowering therapy with a PCSK9 inhibitor had reduced the burden of atherosclerotic cardiovascular disease to achieve LDL-c goals in high-risk FH patients with ACS in a clinical practice setting [7]. Few FH patients received intensive combined lipid-lowering treatment containing a PCSK9 inhibitor in the current study. We are considering that PCSK9 inhibitor could not be widely applied to all ACS patients with FH as soon as possible because of economic costs and unavailability conveniently at that time in western Chinese resource-limited settings. Therefore, FH patients in general were less likely to achieve the target levels of LDL-c recommended by the latest guidelines [1, 15, 16].

With regard to the clinical outcomes, we found that MACCE occurred more often among patients with FH, which was consistent with previous studies [11–13, 33]. Wang et al [11] reported that FH was an independent risk factor for MACCE in young patients after a coronary event during long-term follow-up. It is necessary to optimize lipid-lowering treatment of patients with FH after a coronary event. Akihiro Takasaki et al [13] found that the prevalence of FH in ACS patients from Mie Prefecture was similar to that found in another, multidistrict registry from Japan. Among ACS patients, the short-term incidence of MACCE was not significantly different between patients with and without FH in this study population. Tada et al [12] found that attainment of the LDL-c target was associated with better prognosis in patients with FH. However, the attainment rate is currently inadequate among Japanese individuals. In our study, we found that male sex, smoking, elevated TC, non-HDL-c level, and Lp(a) level were independent risk factors for the occurrence of MACCE among FH patients after univariate and multivariable logistic analyses. According to the Kaplan–Meier curve analysis, patients in the FH group had significantly lower probability of survival than those in the unlikely FH group, and patients with high LDL-c (≥ 1.8 mmol/L) had significantly lower probability of survival than those with low LDL-c (< 1.8 mmol/L) at the 12-month follow-up visit. However, no significant difference was observed between patients with LDL-c levels ≥ 1.4 mmol/L and with < 1.4 mmol/L at the 12-month follow-up visit by using Kaplan–Meier survival analysis. This association between FH clinical diagnosis and MACCE was independent of conventional risk factors. These patients’ high levels of LDL-c may be one of the reasons for their drug-related adverse outcomes. In addition, even after a coronary event, the rate of high-dose statin therapy in FH patients was inadequate in this study. Though some patients with FH were treated with high-dose statins combined with ezetimibe, some of them could still not attain the desirable targets of LDL-c levels. For these patients, novel lipid-lowering drugs such as PCSK9 inhibitors are expected to further decrease LDL-c concentrations and improve cardiovascular outcomes.

### Limitations

Several limitations of our study must be addressed. First, this was a retrospective observational design, and genetic testing was not performed in all the participants to confirm their FH phenotype. Second, the family history of elevated LDL-c was not available for the present study, so we might have underestimated the prevalence of FH in this population. Third, relatively few patients were included in this study, particularly FH patients. The sample size might limit the statistical power of our results. Thus, our findings need to be confirmed in a larger study. Finally, this was a single-site analysis, so our findings might not reflect the general population and should be further investigated in a larger prospective cohort study.

## Conclusions

The prevalence of clinically definite or probable FH was 4.3% and that of possible FH was 10.6% in enrolled patients with ACS. Although the FH phenotype was associated with an increased risk of MACCE, we found that an inadequate high-intensity statins therapy was prescribed to high-risk patients in this current study and a high proportion of patients with this condition were found not to achieve the target LDL-c levels as defined by the current practice guidelines after lipid-lowering therapies containing statins. Thus, it is important to optimize the lipid-lowering treatment for FH patients with ACS after a coronary event.

### Electronic supplementary material

Below is the link to the electronic supplementary material.

Supplementary Material 1

Supplementary Material 2

Supplementary Material 3

## Data Availability

The datasets used and analyzed during the current study are available from the corresponding author on reasonable request.
