# Supplementary material for: Prevalence, clinical features and prognosis of familial hypercholesterolemia in Chinese Han patients with acute coronary syndrome after a coronary event: a retrospective observational study
Source: BMC Cardiovasc Disord. 2024 Mar 5;24:144. doi: 10.1186/s12872-024-03803-4 (PMC10913252; doi:10.1186/s12872-024-03803-4)
Supplement: Supplementary file 1 — Supplementary Material 1 [file 12872_2024_3803_MOESM1_ESM.docx]

**Supplemental table 1 DLCN diagnostic criteria for FH**

| **Criteria** | | **Points** |
| --- | --- | --- |
| **Family history** | |  |
|  | First-degree relative with known premature coronary (men aged <55 years; women <60 years) or vascular diseases, or first-degree relative with known LDL-c above the 95^th^ percentile | 1 |
|  | First-degree relative with tendinous xanthomata and/or arcus cornealis or children aged <18 years with LDL-c above the 95^th^ percentile | 2 |
| **Clinical history** | |  |
|  | Patients with premature CAD (men aged <55 years; women <60 years) | 2 |
|  | Patients with premature cerebral or peripheral vascular diseases (men aged <55 years; women <60 years) | 1 |
| **Physical examination** | |  |
|  | Tendinous xanthomata | 6 |
|  | Arcus cornealis before age 45 years | 4 |
| **LDL-c levels (without treament)** | |  |
|  | LDL-c ≥ 8.5mmol/L (≥325mg/dL) | 8 |
|  | LDL-c 6.5-8.4 mmol/L ( 251-325mg/dL) | 5 |
|  | LDL-c 5.0-6.4 mmol/L ( 191-250mg/dL) | 3 |
|  | LDL-c 4.0-4.9 mmol/L ( 155-190mg/dL) | 1 |
| **DNA analysis** | |  |
|  | Functional mutation in the *LDLR, apoB* or *PCSK9* genes | 8 |
| **A ‘definite’ FH diagnosis requires > 8 points**  **A ‘probable’ FH diagnosis requires 6-8 points**  **A ‘possible’ FH diagnosis requires 3-5 points** | | |

DLCN: Dutch Lipid Clinic Network

FH: familial hypercholesterolaemia

LDL-c: low-density lipoprotein cholesterol

CAD: Coronary artery disease
