# Supplementary material for: Prevalence, clinical features and prognosis of familial hypercholesterolemia in Chinese Han patients with acute coronary syndrome after a coronary event: a retrospective observational study
Source: BMC Cardiovasc Disord. 2024 Mar 5;24:144. doi: 10.1186/s12872-024-03803-4 (PMC10913252; doi:10.1186/s12872-024-03803-4)
Supplement: Supplementary file 2 — Supplementary Material 2 [file 12872_2024_3803_MOESM2_ESM.docx]

**Supplemental table 2 Covariates in a multivariable model**

|  |  |  |  |  | **95%CI for Exp(B)** | |  |
| --- | --- | --- | --- | --- | --- | --- | --- |
|  | **B** | **S.E.** | **Wald** | **Exp(B)** | **lower** | **upper** | ***P* value** |
| **Without FH** | -2.214 | 0.499 | 19.675 | 0.109 | 0.041 | 0.291 | <0.001 |
| **Male** | 1.178 | 0.465 | 6.428 | 3.249 | 1.307 | 8.078 | 0.011 |
| **Smoking** | 0.785 | 0.348 | 5.094 | 2.192 | 1.109 | 4.335 | 0.024 |
| **TC levels** | 2.551 | 1.102 | 5.360 | 12.823 | 1.479 | 111.171 | 0.021 |
| **Lp(a)** | 0.012 | 0.005 | 4.862 | 1.012 | 1.001 | 1.022 | 0.027 |
| **Non-HDL-c** | -1.985 | 0.932 | 4.534 | 0.137 | 0.022 | 0.854 | 0.033 |
| **Constant** | -2.682 | 2.785 | 0.928 | 0.068 |  |  | 0.335 |

FH: familial hypercholesterolemia

TC: total cholesterol

Lp(a): lipoprotein (a)

Non-HDL-c: non high-density lipoprotein cholesterol
