# Supplementary material for: Prevalence, clinical features and prognosis of familial hypercholesterolemia in Chinese Han patients with acute coronary syndrome after a coronary event: a retrospective observational study
Source: BMC Cardiovasc Disord. 2024 Mar 5;24:144. doi: 10.1186/s12872-024-03803-4 (PMC10913252; doi:10.1186/s12872-024-03803-4)
Supplement: Supplementary file 3 — Supplementary Material 3 [file 12872_2024_3803_MOESM3_ESM.docx]

**Supplemental table 3 Outcomes of all patients at 12-month follow-up visit**

|  |  | **The diagnostic probability of FH phenotype** | | |  |  |
| --- | --- | --- | --- | --- | --- | --- |
| **Variable** | **All** | **Definite/Probable** | **Possible** | **Unlikely** | **χ2/U** | ***P* value** |
| **Number, n(%)** | 531(100%) | 15(2.82%) | 56(10.55%) | 460(86.63%) |  |  |
| **LDL-c<1.8 mmol/L, n(%)** | 350(65.91%) | 2(13.33%) | 25(44.64%) | 323(70.22%) | 33.527 | **<0.001** |
| **LDL-c<1.4 mmol/L, n(%)** | 167(47.58%) | 1(6.67%) | 7(12.5%) | 159(34.56%) | 15.672 | **<0.001** |
| **MACCE, n(%)** | 72 (13.56%) | 6(40%) | 24(42.86%) | 42(9.13%) | 57.656 | **<0.001** |

FH: familial hypercholesterolemia

LDL-c: low-density lipoprotein cholesterol

MACCE: major adverse cardiovascular and cerebrovascular events
